# Raman Spectroscopy Discloses Altered Molecular Profile in Thyroid Adenomas

**DOI:** 10.3390/diagnostics11010043

**Published:** 2020-12-29

**Authors:** Armida Sodo, Martina Verri, Andrea Palermo, Anda Mihaela Naciu, Marialuisa Sponziello, Cosimo Durante, Michael Di Gioacchino, Alessio Paolucci, Alessandra di Masi, Filippo Longo, Pierfilippo Crucitti, Chiara Taffon, Maria Antonietta Ricci, Anna Crescenzi

**Affiliations:** 1 Department of Sciences, University Roma Tre, 00146 Rome, Italy; armida.sodo@uniroma3.it (A.S.); michael.digioacchino@uniroma3.it (M.D.G.); ale.paolucci3@stud.uniroma3.it (A.P.); alessandra.dimasi@uniroma3.it (A.d.M.); mariaantonietta.ricci@uniroma3.it (M.A.R.); 2 Pathology Unit, Campus Bio-Medico University Hospital, 00128 Rome, Italy; m.verri@unicampus.it (M.V.); c.taffon@unicampus.it (C.T.); 3 Unit of Endocrinology and Diabetes, Campus Bio-Medico University, 00128 Rome, Italy; a.palermo@unicampus.it (A.P.); a.naciu@unicampus.it (A.M.N.); 4 Department of Translational and Precision Medicine, Sapienza University of Rome, 00185 Rome, Italy; marialuisa.sponziello@uniroma1.it (M.S.); cosimo.durante@uniroma1.it (C.D.); 5 Unit of Thoracic Surgery, Campus Bio-Medico University Hospital, 00128 Rome, Italy; filippo.longo@unicampus.it (F.L.); p.crucitti@unicampus.it (P.C.)

**Keywords:** thyroid, follicular adenoma, follicular carcinoma, molecular markers, Raman spectroscopy, mutational analysis, immunohistochemistry

## Abstract

Follicular patterned nodules are sometimes complex to be classified due to ambiguous nuclear features and/or questionable capsular or vascular invasion. In this setting, there is a poor inter-observer concordance even among expert pathologists. Raman spectroscopy was recently used to separate benign and malignant thyroid nodules based on their molecular fingerprint; anyway, some histologically proved follicular adenomas were clustered as having a characteristic profile of malignant lesions. In this study, we analyzed five follicular thyroid adenomas with a malignant spectroscopic profile compared to five follicular adenomas with a benign Raman spectrum in order to assess possible molecular differences between the two groups. Morphological, immunohistochemical, and molecular analyses evidenced expression of malignancy-associated proteins in four out of five malignant clustered adenomas. The remaining malignant clustered adenoma showed a *TSHR* mutation previously associated with autonomously functioning follicular carcinomas. In conclusion, thyroid follicular adenomas are a group of morphologically benign neoplasms that may have altered the mutational or expression profile; cases of adenomas with altered immunophenotype are recognized as showing a profile associated with malignancy by Raman spectroscopy. This correlation warrants a more extensive evaluation and suggests a potential predictive value of spectroscopic assessment in recognizing characteristics associated with tumor progression in follicular thyroid neoplasms.

## 1. Introduction

Follicular patterned thyroid lesions are a diagnostic dilemma both at cytological and histological examinations. Microfollicular cells groups are the hallmark of cytological samples obtained by fine needle aspiration (FNA) from adenomas (FA), from follicular carcinomas (FC), or sometimes from follicular variant of papillary carcinomas (FV-PTC). Such altered architectural pattern is known as the “gray area” of thyroid cytology, due to the technical difficulty to achieve a definitive diagnosis, prior to surgery. Consequently, most patients undergo diagnostic surgery, with a definitive malignancy diagnosis ranging from 11% to about 47% [1]. In addition, ultrasound (US) investigations of follicular nodules do not result useful for patient’s management, because of FA, FC, and FV-PTC may show quite similar features. On the histological ground, follicular patterned nodules are sometimes complex to be classified due to ambiguous nuclear features, such as clearing or grooves, and questionable capsular or vascular invasion. In this setting, there is a poor inter-observer concordance even among expert pathologists, thus the last edition of WHO classification [2] of tumors of endocrine organs recognizes two subcategories of follicular nodules with uncertain malignant potential. Ancillary tests have been proposed to improve the diagnosis of follicular patterned lesions. Immunohistochemistry is an automatized analysis available in a large number of laboratories. The combination of gal-3, CK19, and HBME-1 seems the most efficient and informative marker panel reaching the greatest specificity (97%) and sensitivity (95%) for the diagnosis of PTC, while TPO membrane staining is usually negative in carcinomas regardless of their histological type [3]. In the contest of follicular nodules, Galectin 3 was proposed as a screening test-method for the preoperative characterization of indeterminate thyroid nodules in different clinical settings; moreover, it is the only test for which methods and reagents for GAL-3-ICC have been standardized for clinical use [4]. Today, these tests are not recommended in routine clinical use since altered phenotypes were sometimes reported in histologically benign cases and the significance of these alterations is still debated. Molecular analysis gives little support in differentiating benign from malignant follicular patterned lesions [5,6]. In these cases, the reported genetic alterations usually involve mutations in RAS genes, whereas p.V600E missense mutation in *BRAF*, which is a typical hallmark of PTCs, has not been detected [7]. The overall genetic profile of FA is similar to that of FC. A few recent studies reported specific genomic features of adenomas and adenomatoid nodules. In a cohort of 259 adenomatoid nodules, the authors identified mutually exclusive *SPOP* p.P94R, *EZH1* p.Q571R, and *ZNF148* mutations in 24.3% of them [7]. Jung et al. evaluated the tumor mutational burdens of FA and FC, reporting that FA and FC genomes show comparable levels of mutations in terms of number, sequence composition, and functional consequences (potential driver mutations) of mutations. They hypothesized that the development of FA requires early genetic alterations (initiation events) such as *RAS*, *EZH1*, *TSHR*, and *EIF1AX* mutations [8]. Additional genetic alterations (progression events), such as mutations in *NRAS* and *HRAS* genes, p.K601E missense mutation in *BRAF*, and copy loss on chromosome 22q, seem to contribute to the progression of FA to FC. Finally, complex gene-expression profiles have been observed in immortalized cells lines established from FA and FC. In this study, the authors identified a cluster of 14 genes, including *LGALS3*, *BCL2L1*, and *TIMP1*, that accurately differentiated these lesions [9]. Other molecular alterations such as HGF/c-met signal expression and cellular distribution in thyroid tumors are currently under evaluation with a 20% expression in FA compared with 100% expression in PTC and 0% in FC [10]. Nevertheless, there is no clear molecular profile suitable to identify FA and to differentiate this lesion from the malignant counterpart. Most importantly, there is only one pathway hypothesis for the progression from FA to FC, which still requires confirmatory studies [8]. Moreover, there is no evidence about a possible malignant potential in lesions morphologically diagnosed as adenomas.

In the last years, Raman spectroscopy (RS) has been used to characterize a variety of biological samples, and in particular, to differentiate between healthy and neoplastic tissues [11,12,13]. RS is based on the inelastic scattering of light from a molecular system. Thus, it can provide information on biological structures, even in complex systems, and may be used to integrate the traditional tools used for cancer diagnosis.

In 2016, for the first time, our group showed that RS is able to discriminate, with high accuracy, between healthy and neoplastic thyroid tissues [14]. More recently, we have identified the molecular fingerprints of thyroid neoplasms [15]. This has allowed to identify, by statistical signal analysis, four clusters, corresponding to healthy/benign, FC, PTC, FV-PTC tissues, among a population of 46 histological thyroid samples. In this study, we have also singled out five follicular thyroid adenomas with a spectroscopic profile that fall in the cluster of malignant lesions. These findings deserve further investigation, stimulating an in-depth examination by different approaches such as immunohistochemical profile and mutational analysis. Herein, we analyzed ten histologically diagnosed adenomas, including those clustered as malignant by RS compared with five adenomas clustered as benign, in order to possibly identify molecular alterations related to ongoing malignant transformation. Indeed, recognizing pathways of malignant progression could allow discriminating non-evolving benign tumors from those requiring surgical management, despite the identical morphology.

## 2. Materials and Methods

### 2.1. Ethics Statement

The study was approved by the Ethical Committee of the UCBM (prot. 33.15 TS ComEt CBM and prot. 31/19 PAR ComEt CBM, 29 July 2019). Enrolled patients signed the informed consent before surgery. Their data were recorded with an anonymous ID code in the software database of the Pathology Unit of the “Campus Bio-Medico” University of Rome. All experiments were performed in full accordance with the principle of Good Clinical Practice (GCP) and the ethical principles contained in the current version of the Declaration of Helsinki.

### 2.2. Thyroid Tissue

From September 2019 to April 2020, patients afferent to the Endocrinology Unit of Campus Bio-Medico for thyroid nodular pathology and receiving an indication to surgery were enrolled in the study. After the submission and signing of informed consent, these patients underwent total thyroidectomy or lobectomy at the surgical unit of the same institution. The removed specimens were immediately submitted unfixed to the pathology unit in an appropriately labelled container. An experienced pathologist valued the gross anatomy of the samples and cut a tissue slice of about 1 cm wide × 1 cm length × 3 mm of thickness, including both healthy and neoplastic areas and avoiding surgical margins. The slice was snap frozen on a metallic cold-plate inside a cryostat. Firstly, a five-micron section was stained with haematoxylin/eosin to confirm the presence of healthy and neoplastic tissue. Closely to the check, four 30 microns thick consecutive cryostatic sections were cut, collected on separate slides, and stored at −80 °C until the Raman evaluation. For diagnostic purpose, the residual slices were defrosted, formalin fixed, and paraffin embedded with the paired surgical samples for definitive histology. Final diagnosis was reported in agreement with the current edition of WHO classification of endocrine tumors [2]. Among the analyzed tissues, ten follicular patterned capsulate lesions, with clear-cut morphological criteria for diagnosis of FA, were included in this study.

### 2.3. Morphological Assessment

Two pathologists (AC, CT) with special experience in thyroid diseases evaluated each case in blind and recorded four different parameters: growth pattern, thyrocytes morphology, Non Invasive Follicular Thyroid neoplasm with Papillary-like nuclear features (NIFTP) score, and fibrous capsule. Growth pattern was reported as follicular, microfollicular, macrofollicular, trabecular, and solid. Thyrocytes were divided in usual morphology and Hurthle cell type. NIFTP score was assigned in agreement with the published scoring scheme [16]. Fibrous capsule was classified as thin, thick, or irregular.

### 2.4. Immunohistochemistry

Following the diagnostic report, a representative slide was selected from each adenoma case. Four-micron paraffin sections were cut, dewaxed, hydrated, and submitted for immunohistochemistry. Immunohistochemical analysis with antibodies for Galectin3 (Cell Marque/SIGMA-ALDRICH, Monoclonal Mouse Antibody, Clone 9C4, Dilution 1:25), CK19 (Dako, Agilent, Monoclonal Mouse Antibody, Clone RCK108, Prediluted), CD56 (Dako, Agilent, Monoclonal Mouse Antibody, Clone 123C3, Dilution 1:100), and HBME1 (Dako, Agilent, Monoclonal Mouse Antibody, Clone HBME-1, Dilution 1:50) was performed in each case using an automatized immunostainer (Omnis, Agilent).

### 2.5. Raman Analysis

Unpolarized Raman spectra were collected by a Renishaw InVia Micro-Raman spectrometer. This is equipped with a solid-state diode laser source at 532 nm with a nominal output power of nearly 60 mW and a Leica DM2700 M confocal microscope, with a 50X LWD and a 100X objectives. A holographic edge filter determines the high-contrast rejection for the elastically scattered light, and a diffraction grating (1800 grooves/mm) provides a spectral resolution of about 1 cm^−1^. Scattered photons are detected by a Peltier cooled CCD (1024 × 256 pixel). The laser power at the sample was set by neutral density filters, to prevent photo-damage of tissues. The spot size was set to a few microns. Spectra were collected in the extended scan mode, covering the 100–3800 cm^−1^ wavenumber range. Wire, LabSpec, MatLab, and Origin software were used to collect, refine, and analyze the raw spectra.

### 2.6. Cluster Analysis of Raman Spectra

An agglomerative hierarchical clustering analysis (AHCA) was performed on the collected Raman spectra, after preliminary data treatment (e.g., background subtraction, smoothing, etc.). AHCA measures the Euclidean distance among the spectra, based on their similarity/dissimilarity and returns a dissimilarity matrix of all spectra. In the present instance, we used the Euclidean distance, to maximize the similarities among the spectra, along with the complete-linkage algorithm, in order to enhance differences among the data clusters. This algorithm pairs the most similar spectra (those showing the lowest dissimilarity value in the dissimilarity matrix) and then searches the largest distance between such pairs and the rest of the data. The process iteratively proceeds until a classification of the spectra into well-separated groups is obtained and visualized as a dendrogram.

### 2.7. Molecular Analysis

Each sample was tested for a previously established dual-component molecular assay that includes next-generation sequencing (NGS)-based detection of mutations in 23 thyroid cancer-related genes and digital polymerase chain reaction (dPCR) evaluation of the expression levels of an microRNA strongly associated with thyroid cancer (miR-146b-5p) [17]. Molecular markers were selected with the aim of identifying the minimum number of markers that can distinguish benign thyroid nodules from malignant thyroid nodules. Five paraffin sections 10 micron thick were cut from the representative block of each adenoma case, previously used for immunohistochemistry. RecoverAll Total Nucleic Acid Isolation Kit (Thermo Fisher Scientific, Waltham, MA, USA) was used to simultaneously isolate DNA and RNA, following the manufacturer’s instructions. Nucleic acid concentrations were measured using fluorescence-based Qubit^®^ quantification assays for DNA and RNA (Thermo Fisher Scientific, Waltham, MA, USA). Briefly, for mutation detection, two amplification-based libraries were prepared with 15 ng of DNA and 10 ng of RNA using two custom thyroid-specific panels (DNA and RNA panels), the Ion AmpliSeq™ Library Kit Plus and Ion Xpress™ Barcode Adapter 1–96 Kit according to the manufacturer’s recommendations (Thermo Fisher Scientific, Waltham, MA, USA) [17,18,19]. Libraries were clonally amplified on the Ion One Touch2 System, and subsequently sequenced on the Ion Gene Studio S5 (Thermo Fisher Scientific, Waltham, MA, USA) with an Ion 540™ Chip (Thermo Fisher Scientific). Data were analyzed with Torrent Suite v.5.10 software (Thermo Fisher Scientific, Waltham, MA, USA). Annotation of genetic variants was performed with wANNOVAR web server and analysis of gene fusions with the IonReporter 5.12 software (Thermo Fisher Scientific, Waltham, MA, USA) [17,18].

Expression levels of the miRNA-146b-5p were evaluated using dPCR on a QuantStudio 3D Digital PCR Instrument (Thermo Fisher Scientific, Waltham, MA, USA) as previously described [17]. The molecular test was considered positive when miRNA expression levels in the analyzed specimen exceeded the previously calculated cut-off value [17].

## 3. Results

Baseline characteristics. The study population encompassed ten patients with follicular adenoma of thyroid gland, four male and six female, of age ranging from 27 to 78 years. Diameters of the nodules ranges from 8 to 38 mm (21.5 ± 10.46 mm mean ± SD) with an US risk ranging from 3 to 4 in agreement with the Thyroid imaging reporting and data system (TIRADS) [20]. Seven patients had previously received fine needle aspiration biopsy with a cytological report of benign (TIR2) in two cases, low risk indeterminate lesion (TIR3A) in three cases, and high risk indeterminate lesions (TIR3B) in two cases, based on the Italian Consensus for thyroid cytology [21].

### 3.1. Morphological Evaluation

The morphological analysis revealed a follicular pattern of growth with various intermingling of micro, normo, or macrofollicular arrangement. No trabecular or solid pattern were observed. Five out of ten cases show near total Hurthle cells population. NIFTP score ranges from 0 to 1 in nine out of ten cases; the remaining cases have focal nuclear modification of score 2. Capsules are thin in seven cases and irregular in three.

### 3.2. Raman Clustering

In a previous study, we applied RS augmented by cluster analysis to investigate histological samples from human thyroid [15]. This approach allowed us to classify each Raman spectrum into homogeneous groups, characterized by the presence of bands from carotenoids and cytochrome c, within the fingerprint region (600–1800 cm^−1^). This allowed to distinguish healthy tissues from cancerous ones, and to discriminate for the first time among papillary (PTC), FV-PTC, and FC.

**Figure 1:**
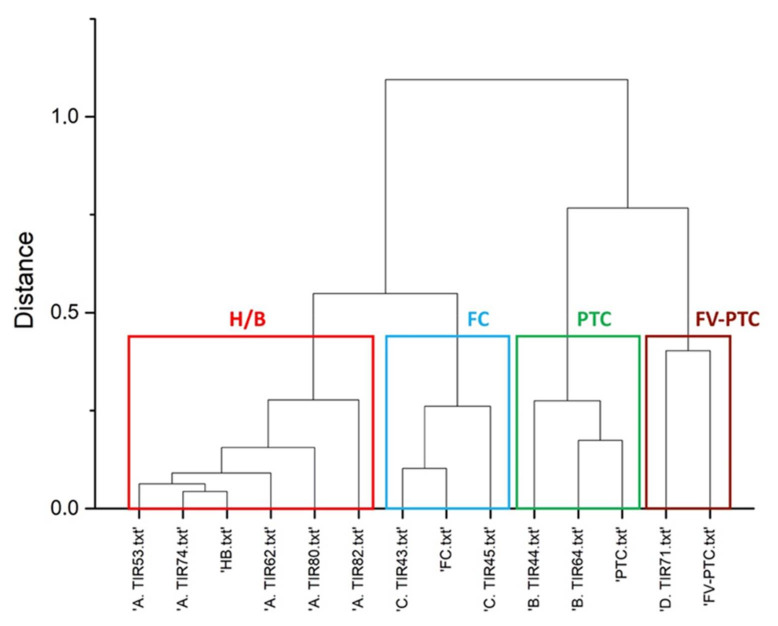
Agglomerative hierarchical clustering analysis (AHCA) dendrogram of the ten adenoma samples investigated. Notice that: 5 of them fall within the healthy cluster (labelled H/B, in red, A), while the others are spread among the three cancer categories, namely follicular carcinoma, labelled FC (blue, C), papillary carcinoma, labelled PTC (green, B)), and follicular variant of papillary carcinoma, labelled FV-PTC (brown, D). HB.txt, FC.txt, PTC.txt, and FV-PTC.txt indicate the reference spectrum for each category.

The results, obtained by comparing the Raman spectra of these samples, classified as adenoma by histological diagnosis, with the reference samples used in the AHCA analysis, are reported in Figure 1. The analysis identified five histologically proved adenomas as cancer cases among the ten investigated. In particular, Raman clustering analysis classified TIR 44 and TIR64 as PTC; TIR71 as FV-PTC; TIR43 and TIR45 as FC; and TIR53, TIR62, TIR74, TIR80, TIR82 as healthy/benign. Thus, all the ten samples were sent for a deeper immunohistochemical and molecular investigation, along the lines described below, in order to clarify if these samples are evolving towards a cancerous diagnosis.

### 3.3. Immunohistochemical Assessment

**Figure 2:**
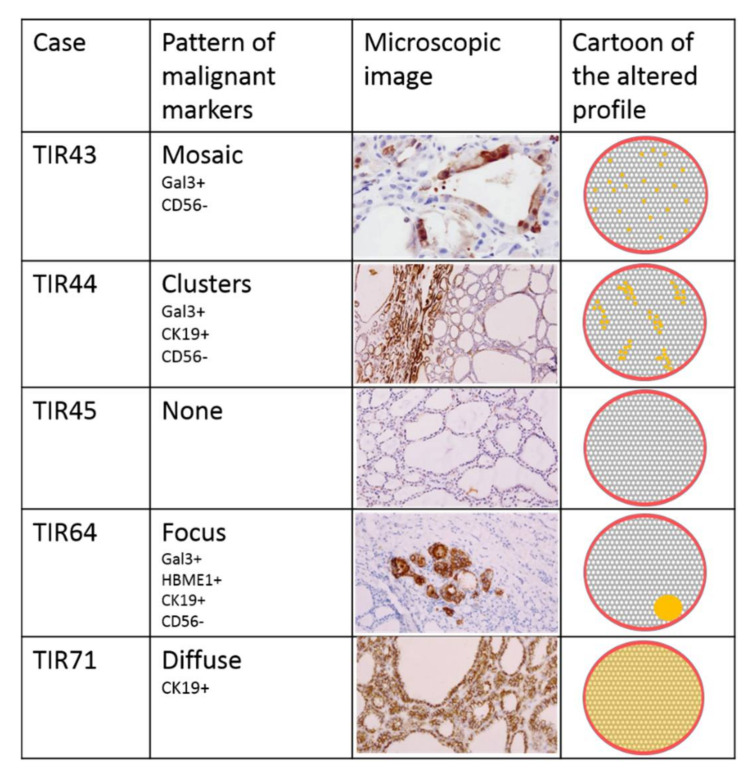
Five cases that dropped in the malignant Raman clusters: four of them show altered expression of immunohistochemical markers, although with different distribution on the samples. (+ positive reaction; - negative reaction)The cartoons on the right-hand panels model different patterns of markers expression.

**Figure 3:**
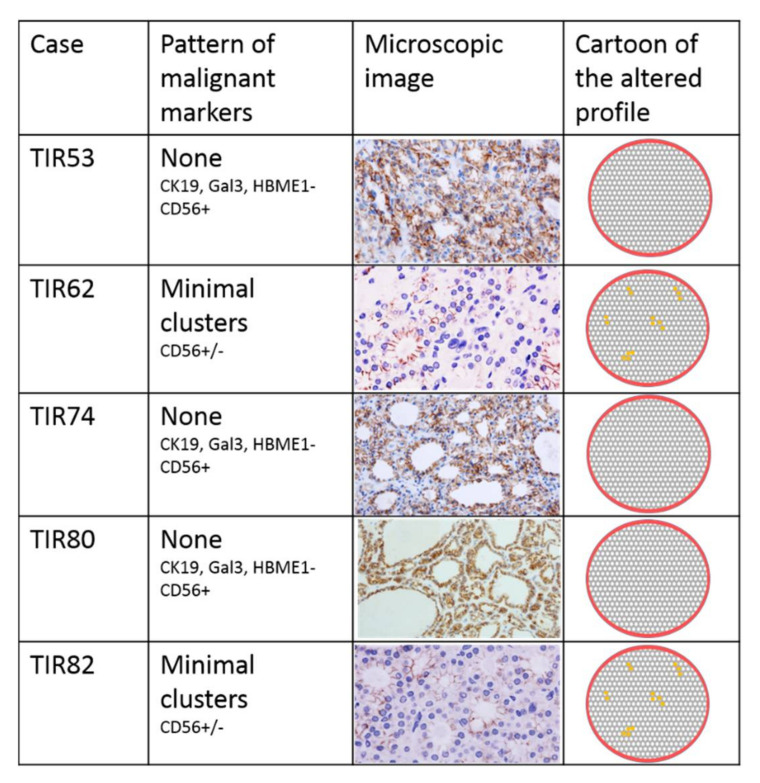
Five cases that dropped in the benign Raman clusters: none of them show expression of proteins acquired during neoplastic transformation; two out of five show minimal loss of CD56 (see microscopic images) (+ positive reaction; - negative reaction). The cartoons on the right-hand panels model different patterns of markers expression.

Immunohistochemistry has revealed altered expression of markers in six cases with different pattern: mosaic alteration was referred to positive cells intermixed with negative ones; cluster pattern was referred to groups of positive cells in a negative background; focus pattern was referred to a single area of altered markers; diffuse pattern means that all cells showed positive profile for at least a marker. The altered immunophenotype was observed in four of five adenomas with malignant Raman clustering (Figure 2). Two cases out of five adenomas with benign Raman clustering showed a minimal loss of CD56 staining with cluster pattern; none of the RS benign nodules showed expression of proteins correlates to malignant transformation (Figure 3).

### 3.4. Molecular Analysis

In order to better clarify the molecular events that characterize the 10 follicular adenomas, we profiled them with a dual component molecular assay: an amplification-based NGS assay targeting 23 thyroid cancer-related genes and a dPCR assay evaluating miR-146b-5p expression levels [17]. NGS-based analyses revealed four gain-of-function mutations of *TSHR* (p.I486F, COSM26450; p.D633Y, COSM26419; p.S505N, rs121908876; p.D633E), occurring in the transmembrane domain of the relative protein. In addition, one out of the four mutated cases harbored a second variant in *EIF1AX* gene (p.R13L; AF:24%; COSM6908853). All samples were negative for miRNA expression analysis. Clinical characteristics, morphological features, molecular data, and Raman clustering are summarized in Table 1.

## 4. Discussion

Our previous study using Raman spectroscopy to evaluate the biochemical profile of thyroid lesions [15] demonstrated that RS is able to discriminate malignant thyroid lesion from benign nodule and healthy thyroid parenchyma with an accuracy of 90%. Of relevance, Raman spectroscopy also separated malignant clustered cases in follicular carcinomas and papillary carcinomas. In the same study, we found five cases histologically diagnosed as adenomas that were reclassified as malignant in the Raman clustering, in particular, two were reported as PTC, one as FV-PTC, and two as FC. To better understand the molecular changes underpinning this discrepancy between morphology and spectroscopy, we performed an extensive evaluation of ten thyroid nodules, submitted for surgery with final histological diagnosis of follicular adenoma. The present study includes five nodules clustered as benign by RS and five clustered as malignant (Figure 1). The first step was the morphological assessment of cytological and histological parameters. This evaluation did not reveal differences between benign and malignant clustered cases as far as both their cytological aspect (Hurthle versus usual thyrocytes) and histological pattern (capsular profile, follicular architecture). Differences between the two groups were not visible using the nuclear NIFTP score in 9/10 cases; only in one case (TIR64), clustered as malignant, a focus of the NIFTP score 2 of about 1 mm, was visible inside the adenoma proliferation. Therefore, morphological assessment does not seem informative enough to discriminate among these lesions. In addition, clinical and ultrasound parameters such as age, gender, nodule diameter, and TIRADS classification did not show correlation with the RS clustering. The second step was the immunohistochemical assessment for expression of cellular proteins that are acquired (Gal3, HBME1, CK19) or lost (CD56) during neoplastic transformation in thyroid oncogenesis. This evaluation revealed an altered immunophenotype in four out of five of the malignant clustered adenomas (TIR43, TIR44, TIR64, and TIR71) with a different pattern of expression, including lowering of normal proteins and/or expression of the malignancy-associated ones. No rise of malignancy-associated proteins was observed in the adenomas clustered as benign, while focal loss of expression of CD56 was noted in two (TIR62 and TIR82) out of five cases. This last finding may be not significant, being focal and limited to a single protein loss. The specific pattern of the immunohistochemical changes shows variations in the protein expression profile among the malignant clustered cases (Figure 2), thus supporting a heterogeneous background of the mRNA modulation in these cases. Anyway, the prevalence of altered immunophenotypes in the malignant clustered cases, suggests that some differences exist between the two groups (namely malignant and benign cluster) and that a remodeling of the proteins’ transcription occurs in the adenomas recognized as malignant by RS, supporting the hypothesis that RS might be predictive of altered phenotype in some follicular adenomas. Interestingly, in 3 on 5 malignant clustered cases, we observed a combination of altered immunohistochemical markers that are considered to reach high specificity and sensitivity for the diagnosis of carcinoma. Such cases are morphologically indistinguishable from the benign clustered ones.

Targeted NGS of the ten adenomas revealed a high prevalence of gain-of-function mutations of the *TSHR* gene (40%). TSHR is a G-protein coupled membrane receptor with a pivotal role in physiological function and pathophysiology of benign and malignant thyroid diseases. While activating somatic mutations of *TSHR* have been found in up to 84% of toxic thyroid nodules, they are rare in malignant thyroid tumors [22]. Individual malignant cases were reported including autonomously functioning follicular thyroid carcinomas, hyper-functioning Hurthle carcinoma, and papillary thyroid carcinoma. However, if and how these mutations can contribute to the development of thyroid carcinoma is still debated. Among the *TSHR* mutations we found, the p.I486F has been previously associated with an autonomously functioning follicular carcinomas [23] whereas p.D633Y mutation has been reported in a toxic metastasizing FC [24], an Hurthle cell carcinoma, and in a pediatric PTC [25]. Notably, the *TSHR* p.I486F mutation occurs in the TIR45 case that RS classified in the malignant cluster of FC (Table 1).

Out of *TSHR* mutations, we have not found any relevant differences in the molecular assessment between benign and malignant RS clustered cases; in particular, no mutations were identified in the main gene drivers (*BRAF* and *RAS*). These data are in agreement with a complete independence of the Raman clustering from the mutational profile in thyroid carcinomas [15]. Moreover, no differences were found when analyzing RNA fusions, nor miRNA expression. However, the presence of molecular alterations other than those analyzed cannot be ruled-out.

## 5. Conclusions

In conclusion, thyroid follicular adenomas are a group of clinically benign neoplasms that may have altered mutational or expression profile; the diagnostic impact of these results is still unknown. Cases with altered immunophenotype are categorized as malignant by RS; this correlation deserves a more extensive evaluation aimed at understanding the potential predictive value of spectroscopic assessment in recognizing characteristics associated with tumor progression in follicular thyroid neoplasms.

## Figures and Tables

**Table 1:** Patients with follicular adenoma. Clinical features and morphological characteristics of the nodules. Legend: * Focus 1 mm PTC; n.a. not available, NGS, next-generation sequencing; dPCR, digital PCR; AF, allele frequency. (+ positive reaction; - negative reaction).

Case		TIR43	TIR44	TIR45	TIR53	TIR62	TIR64	TIR71	TIR74	TIR80	TIR82
**Gender**		F	M	F	F	M	F	M	M	F	F
**Age**		42	63	78	27	52	66	44	39	67	32
**Max Diameter (mm)**		38	30	8	18	15	16	12	38	18	22
**FNA**		TIR3B	n.a.	TIR2	TIR3A	TIR3B	TIR3A	TIR2	TIR3A	n.a.	n.a.
**Clinical** **Features**	**TSH**	1.67	0.9	0.5	0.5	0.004	2.39	0.16	0.6	0.01	0.02
	**TIRADS** **(1,2,3,4,5,6)**	4	3	4	4	3	4	4	3	3	3
**Morphological Features**	**Growth Pattern**	micro/ normo follicular	micro/ macro follicular	micro/ normo follicular	micro/ normo follicular	micro/ macro follicular	micro/ macro follicular	micro follicular	micro/ macro follicular	micro/ macro follicular	micro/ normo follicular
	**Thyrocytes**	Hurtle	Usual	Hurtle	Hurtle	Hurtle	Usual	Usual	Hurtle	Usual	Usual
	**NIFTP score**	1	1	0	0	1	2 *	1	0	0	1
	**Capsule**	Irregular	Thin	Irregular	Thin	Thin	Thin	Thin	Thin	Thin	Irregular
**Immuno-** **Histochemical** **Markers**	**Gal3**	- mosaic	+ cluster	-	-	-	+ focus	-	-	-	-
	**HBME-1**	-	-	-	-	-	+ focus	-	-	-	-
	**CK19**	-	+ cluster	-	-	-	+ focus	+ diffuse	-	-	-
	**CD56**	- mosaic	- cluster	+	+	- cluster	- focus	- cluster	+	+	- cluster
**Molecular** **Analysis** **(NGS + dPCR)**	**SNVs/** **Indels**	Negative	Negative	**TSHR** p.I486F; AF:28%	**TSHR** p.D633Y; AF:39%	Negative	Negative	Negative	Negative	**TSHR** p.S505N AF:25%; **EIF1AX** p.R13L; AF:24%	**TSHR** p.D633E; AF:40%
	**Gene** **Fusions**	Negative	Negative	Negative	Negative	Negative	Negative	Negative	Negative	Negative	Negative
	**miRNA**	n.a.	n.a.	Negative	n.a.	Negative	Negative	Negative	Negative	Negative	Negative
**Raman Cluster**		Malignant FC	Malignant PTC	Malignant FC	Benign	Benign	Malignant PTC	Malignant FV-PTC	Benign	Benign	Benign

## Data Availability

The data presented in this study are available in the article (Table 1).
